# The Major Depressive Disorder Hierarchy: Rasch Analysis of 6 items of the Hamilton Depression Scale Covering the Continuum of Depressive Syndrome

**DOI:** 10.1371/journal.pone.0170000

**Published:** 2017-01-23

**Authors:** Lucas Primo de Carvalho Alves, Marcelo Pio de Almeida Fleck, Aline Boni, Neusa Sica da Rocha

**Affiliations:** 1 Federal University of Rio Grande do Sul, Porto Alegre, Brazil; 2 Hospital de Clínicas de Porto Alegre, Porto Alegre, Brazil; 3 Programa de Pós-Graduação em Psiquiatria e Ciências do Comportamento, Porto Alegre, Brazil; Pennsylvania State University College of Medicine, UNITED STATES

## Abstract

**Objectives:**

Melancholic features of depression (MFD) seem to be a unidimensional group of signs and symptoms. However, little importance has been given to the evaluation of what features are related to a more severe disorder. That is, what are the MFD that appear only in the most depressed patients. We aim to demonstrate how each MFD is related to the severity of the major depressive disorder.

**Methods:**

We evaluated both the Hamilton depression rating scale (HDRS-17) and its 6-item melancholic subscale (HAM-D6) in 291 depressed inpatients using Rasch analysis, which computes the severity of each MFD. Overall measures of model fit were mean (±SD) of items and persons residual = 0 (±1); low χ^2^ value; p>0.01.

**Results:**

For the HDRS-17 model fit, mean (±SD) of item residuals = 0.35 (±1.4); mean (±SD) of person residuals = -0.15 (±1.09); χ^2^ = 309.74; p<0.00001. For the HAM-D6 model fit, mean (±SD) of item residuals = 0.5 (±0.86); mean (±SD) of person residuals = 0.15 (±0.91); χ^2^ = 56.13; p = 0.196. MFD ordered by crescent severity were depressed mood, work and activities, somatic symptoms, psychic anxiety, guilt feelings, and psychomotor retardation.

**Conclusions:**

Depressed mood is less severe, while guilt feelings and psychomotor retardation are more severe MFD in a psychiatric hospitalization. Understanding depression as a continuum of symptoms can improve the understanding of the disorder and may improve its perspective of treatment.

## Introduction

Major depressive disorder (MDD) is one of the most frequent psychiatric disorders both in the community and in psychiatric settings [1]. The World Health Organization presented a report in 2011 estimating that depressive disorders were the second leading cause of years lived with disability [2]. However, several studies show that MDD is a very heterogeneous syndrome [3–6] that is characterized by many different presentations and possibly with different risk factors for its individual symptoms [7,8]. Actually, MDD DSM-V [9] are quite similar to DSM-III criteria released in 1980 [10]; there are only minor changes regarding bereavement [11]. Nonetheless, most experts agree that DSM criteria have been chosen through consensus instead of empirical research [12]. Oostergaard et al (2011) showed that 1,492 different presentations of the same syndrome according the DSM-IV criteria [9] are mathematically possible [13]. Therefore, a particular problem is created when we consider some disorders categorical, while evidence favors a dimensional approach for them; MDD is a major example [14]. Symptoms listed in DSM-V for MDD criteria seem to have a Gaussian distribution in the general population [15], suggesting that it may be a diagnostic convention imposed on a continuum of depressive symptoms [14]. Nevertheless, although there is still no quantitative laboratory measures for the diagnosis and evaluation of severity of MDD, levels of impairment have long been successfully represented in quantitative scales [15].

In this context, with the improvement of modern psychometrics, we are able to identify groups of clinical features that result in a unidimensional and homogeneous syndrome. For example, experienced psychiatrists always considered that MDD could be categorized between a biological, “endogenous” depression–called melancholic depression–and a psychogenic, “exogenous” depression–called non-melancholic depression [16,17]. This perception is supported by controversial evidences that melancholic patients would have genetic and biological determinants; more severe symptomatology; more evidence of neurobiological abnormalities, especially of the hypothalamic-pituitary-adrenal axis; low response to placebo; and superior response to pharmacological and electroconvulsive therapies [5,18–20]. With this concept in mind, Bech et al (1975) found a valid subgroup of six items of the 17-item Hamilton depression rating scale (HDRS-17) (21)(21)(21)[21] that could represent the “core symptoms” of MDD when evaluating melancholic patients by the 17-item HDRS-17 [21]. These six items (which we will now refer as “melancholic features”) are depressed mood, feelings of guilt, work and interest, psychic anxiety, general somatic symptoms, and psychomotor retardation. This subscale has been called the “melancholia subscale”, or HAM-D_6_ [21], and has demonstrated that it can meet the criteria for unidimensionality. It is also clinically meaningful, more psychometrically robust, and less likely to mistake side effects of drugs for improvement or worsening in depression [22,23]. Furthermore, even when applied in non-melancholic patients, the HAM-D_6_ has shown that it can provide larger effect sizes in clinical trials [24] and even a need for fewer study participants to demonstrate superior antidepressant efficacy [25] in comparison to HDRS-17. In addition, according to Bech, the remaining items of HDRS-17 are unspecific symptoms of physiological reaction to stress (9 items) or evaluate suicide risk (2 items) [12].

Systematic review of MEDLINE showed that there are still few studies that have evaluated the HDRS-17 with modern psychometrics [26]. Most of them focused in the assessment of the dimensionality of the scale [27–32], asking, for instance, “what does the Hamilton scale really measure?”[4], suggesting that it could measure more than one single construct. However, little importance has been given to the question “are the items of the scale adequate to measure all the continuum of the severity of depression syndrome?”

In this context, there are already well-established statistical tools to evaluate the properties of each scale item (melancholic features) and to relate them to the severity of the disorder. Rasch analysis is one of the most robust tools available. It computes the probability of patients to score each question related to the items, and with this data, it generates a scalar score of severity for both the patients and the items. Thus, in addition to the important property of evaluation of the dimensionality of the scale, Rasch analysis is able to identify what questions are related to a more severe disorder. That is, what are the melancholic features that appear only in the most depressed patients. Indeed, when HAM-D_6_ was constructed, Bech et al (1981) used Rasch analysis [31], but the method has been improved since then, making it possible to work with items with different numbers of categories [33]. In his original study of HAM-D_6_, Bech showed that work and activities and depressed mood represent the less severe symptoms, while feelings of guilt and psychomotor retardation represent the most severe melancholic features. However, the main focus of Bech studies was to establish a comparison between scales and showing the property of HAM-D_6_ unidimensionality rather than aiming to set up a symptom hierarchy in the evaluation of melancholic depression [32].

There are some useful reasons to attribute a severity value for signs and symptoms that have the properties of unidimensionality. First, we can move away from the view of a single score (sum of items) as a measurement of severity by focusing on the severity value of each sign or symptom. Second, we can evaluate the presence or absence of MDD clinical features as a clinical marker of severity or improvement, respectively. Ultimately, we can understand the MDD syndrome through the clinical importance of each clinical feature instead of a sum of items that may be not part of the same underlying syndrome.

The objective of this study is to demonstrate how each clinical feature measured by HAM-D_6_ are related to the severity of the syndrome and to build a hierarchy of MDD signs and symptoms.

## Material and Methods

### Study Design and Sample Selection

We conducted a cross-sectional study in a sample of severely depressed inpatients who were admitted at the psychiatric unit of the Hospital de Clínicas de Porto Alegre, Porto Alegre, in southern Brazil between May 2011 and October 2013. Written informed consent was obtained from all patients. Comissão Científica e Comitê de Ética em Pesquisa from Hospital de Clínicas de Porto Alegre approved the study. Approval Number: 100265. Hospital de Clínicas is a university general hospital of tertiary care. Its psychiatry unit is within the hospital, and attending patients arise from both the public health system and health insurance plans, since they are both a unit of reference in southern Brazil.

Trained psychiatrists or psychiatry graduate students conducted a Mini-International Neuropsychiatry Interview (MINI) to all psychiatry inpatients at admission. If the MINI fulfilled the criteria for MDD, the same evaluator applied HDRS-17 concurrently. Patients were included if they were 18 years of age or more and were diagnosed with major depression according to the MINI based on the DSM-IV criteria. Patients were excluded from the study based on the following criteria: length of hospital stay was less than 7 days; drug or alcohol addictions or dependence was the main diagnosis at admission; or severity scales could not be obtained. We obtained patients’ demographic characteristics through a questionnaire and from medical records.

### Statistical Analysis

People who collected data from the MINI and HDRS-17 did not participate in the statistical analysis. Demographic data were analyzed with SPSS® v.21, and Rasch analysis was performed for both the HDRS-17 and its subscale HAM-D_6._ using RUMM2020 [34] software. Subjects who had at least 1 missing value in the scales were excluded from the Rasch analysis.

The statistical method of Rasch analysis is described elsewhere [35]. Briefly, the Rasch model allows the assessment of the performance of each individual item (question), rather than the total score, to evaluate the severity of the syndrome. It has two main characteristics: (1) the ability to verify the dimensionality of an underlying syndrome; (2) once the scale is accepted as unidimensional, it turns the raw data (sum of items, i.e., total score) of both the persons and the items in a scalar measure. The Rasch analysis generates a score of severity called the “Rasch score” using logit as unit of measurement, which is based on the probability of the persons to answer each item and generates a scalar (and more precise) variable. This property is valid not only for *persons* but for *items* as well, so we can evaluate the most severe melancholic features of the syndrome. If one item is measuring a more severe intensity of the trait than the other, it means that only the more severe patients will highly punctuate this sign or symptom. With this approach, it is possible to build a “melancholic features hierarchy” (i.e., ordering) that can identify items’ importance considering traits as a continuum.

This is possible because the model is based on an iterative method that computes the probability of scoring any question by relating the person severity *and* the item severity [33]: a higher the severity of the person’s intensity of the trait is associated with the higher the probability to score higher in a severe item (i.e., sign/symptom) [36]. Taking a school test as an example, we can consider the test as a series of questions with different levels of difficulty. The most difficult questions will discriminate the ones with the best performance from the others. Extrapolating to the psychiatry field, Rasch analysis can find which depressive symptoms are scored higher only by the most depressed patients. The severity of the symptom is expressed by the location of the Rasch score (i.e., the higher the number of the location, the more severe the symptom). Likewise, the depression severity of a single person is also expressed by the location of its Rasch score. When a person has the same Rasch score of an item, it means that the person has a 50% probability to endorse that item. This means that patients who have a Rasch score higher than one item are more likely to answer the item correctly than to answer it wrongly.

In contrast with most IRT models (which seek the model that best accounts for the data), Rasch analysis accepts the form of the model and evaluates whether the data fit that form [37]. The Rasch model assumes that the scale is unidimensional, which can be assessed by three main approaches [32,35,38–40]: (a) principal component analysis of the person residuals, with the scale considered unidimensional if the first factor does not account for at least 40% of the variance; (b) independent t tests between persons estimates derived from the highest positive set of items and person estimates derived from the highest negative set of items, considered unidimensional when either less than 5% of t tests are significant, or if the lower bound of the binomial confidence interval overlaps the 5%; (c) if observed data fit to the Rasch model. The latter approach, although not sufficient to determine the dimensionality of the scale, particularly when different dimensions have equal number of items contributed to them [40], is one of the most robust. When data does not fit to the model, it means that the data does not match the mathematically unidimensional framework that the Rasch analysis has constructed; thus, if Rasch model assumptions are violated, we probably need a model with two or more parameters to the persons to describe the data, violating the assumption of unidimensionality [32,41–43].

Three overall fit statistics determine the model fit to Rasch analysis: (a) mean and (b) standard deviation (SD) of the residuals of item-person interaction statistics, distributed as Z statistics (where having a mean of zero and SD of 1 indicates perfect fit to the model), and the (c) χ2 item-trait interaction (of total items), which should be nonsignificant, reflecting the property of invariance across the trait. If χ2 is low and its P value is consequently greater than 0.05, then the null hypothesis is not rejected and the scale is accepted to fit the Rasch model [32]. Chi-Square item-trait interaction, as well as any other statistical test, is also dependent of the sample size. While a small sample size generates unstable results, a large sample may result a significant χ2 value even with small deviation from the Rasch model. Simulation studies show that a sample size of 250 patients may get accurate estimates of item and person locations regardless of the scale targeting [39,44,45]. RUMM2020 software allows specifying an effective sample size in the χ2 formula, with all equations and data remaining the same [46]. Hence, if χ2 P value was <0.05, we computed a new test of fit, using 200 as total sample, to assure that misfit was not only because of the sample size.

RUMM2020 also computes two reliability index of the scale, measuring the internal consistence of it. They are the Cronbach’s alfa, derived from the classical test theory, and the Pearson Separation Index (PSI), constructed with Rasch measurement. Both have similar values in complete data, but the latter works better with missing data [40,46]. Although those index are not a measure of quality of the scale, but of relative reproducibility [47], and dependent of the number of items [12], we reported them, since data are already provided by the software.

## Results

We included a sample of 291 individuals with diagnosis of MDD. Demographic characteristics of the sample are shown in Table 1. For the HDRS-17, only one subject had 1 missing value, and so they were excluded from the analysis. This did not happen to HAM-D_6_, because the missing value was on item 5 of the HDRS-17 (intermediate insomnia), which is not part of the HAM-D_6_. So, the subject was re-included in the analysis of HAM-D_6_.

**Table 1:** Demographic characteristics of the sample.

Characteristic	Subjects (n total = 291)
Sex • *Male*	116 (39.9%)
Mean Age, in years (±SD)	45.72 (±14.49)
Ethnicity • *Caucasian* • *Not Caucasian*	239 (82.1%) 42 (14.4%)
Mean Years of Study (±SD)	9.14 (±4.7)
Previous Psychiatry Hospitalization	187 (64.3%)
Mean Number of Previous Psychiatry Hospitalizations (±SD)	3.19 (±5.21)
Psychosis in the Past	106 (36.4%)
Suicide Attempt in the Past	183 (62.9%)
Presence of Psychotic Symptoms^*^	84 (28.9%)
Mean Depression Score–sum of scores (±SD) • *HDRS-17 (n = 290)* • *HAM-D*_*6*_ *(n = 291)*	22.49 (±6.8) 10.64 (±3.58)

*Evaluated through Mini-International Neuropsychiatric Interview.

Four items of HAM-D_6_ had disordered categories according to Rasch analysis and required further adjustments. The four items of HAM-D_6_ that had disordered thresholds were depressed mood, guilt feelings, work and activities, and psychic anxiety. Depressed mood, guilt feelings, and work and activities were transformed into a 3-category item. Psychic anxiety was transformed into a 4-category item.

### Rasch Analysis

Results of the Rash analysis of HDRS-17 and HAM-D_6_ (already with corrections) are shown in Tables 2 and 3. HDRS-17 had a PSI of 0.660 and a Cronbach’s alpha of 0.693, while HAM-D_6_ had a PSI and Cronbach’s alpha of 0.54 and 0.5. The unidimensionality indexes through principal component analysis of the person residuals were fulfilled to both scales. However, fit to unidimensional Rasch model of HDRS-17 total χ2 was very high, with a p value <0.00001, demonstrating that HDRS-17 does not fit to the unidimensional Rasch Model (below 0.05). When tested with an effective lower sample size (n = 200), total item Chi-Square was 213,46, with a p value of 0,0009, showing that misfit was not due to sample size. Item and person residual means and the SD of HAM-D_6_ had a better fit to the model (i.e., more close to the perfect mean residual–zero–and the perfect SD residual–one). Furthermore, the total χ2 was 56.13 with a p value of 0.19 (above 0.05), demonstrating that the scale fits to the unidimensional Rasch model, even with a higher effective sample size.

**Table 2:** Rasch analysis of HDRS-17.

Hamilton Depression Rating Scale– 17 items						
Item	Location (“severity score”)	Residual^a^	χ2	P Value	Summary of overall measures of model fit statistics	
Depressed Mood	-1.36	-2.04	25.65	0.02	Item fit residual mean (SD)	0.35 (1.4)^b^
Work and Interests	-1.02	0.16	8.82	0.45		
Insomnia, initial	-0.99	-0.55	9.02	0.43		
Genital	-0.91	-0.17	18.82	0.03	Person fit residual mean (SD)	-0.15 (1.09)^b^
Suicide	-0.56	-1.27	18.91	0.02		
Insomnia, intermediate	-0.52	-0.37	7.98	0.54		
Somatic, general	-0.35	0.2	15.76	0.07	% of the 1st principal component of person residuals	14,17%^c^
Insomnia, delayed	-0.28	-0.25	6.64	0.67		
Anxiety, psychic	0.01	-0.67	18.08	0.03		
Somatic, gastrointestinal	0.1	-1.21	33.82	0.00	% t tests (95% CI)	5,5 (0,03–0,09)^d^
Guilt	0.3	0.53	11.96	0.21		
Hypocondriasis	0.31	0.3	7.74	0.56		
Loss of weight	0.71	2.42	21.38	0.01	Total Item χ2	**309.744**^**e**^
Somatic Anxiety	0.98	2.28	39.73	0.00		
Retardation	1.1	2.37	16.1	0.06		
Agitation	1.23	2.54	44.03	0.00	χ2 p value	**<0.00001**^**f**^
Insight	1.23	1.15	5.30	0.8		
				

^a^ Model fit when the value is between -2,5 and 2,5.

^b^ Perfect model fit when mean = 0 and standard deviation (SD) = 1.

^c^ Considered unidimensional when <40%.

^d^ Considered unidimensional when either less than 5% of t tests are significant, or if the lower bound of the binomial confidence interval overlaps the 5%.

^e^ Best model fit when it is a low number.

^f^ Model fit when >0.01, demonstrating the fit to the unidimensional Rasch model.

**Table 3:** Rasch analysis of HAM-D_6_.

Hamilton 6 item—Melancholia Subscale						
Item	Location (“severity score”)	Residual^a^	χ2	P Value	Summary of overall measures of model fit statistics	
Mood	-2.02	-0.79	22.66	0.00	Item fit residual mean (SD)	0.5 (0.86)^b^
Work and Activities	-1.11	0.57	9.02	0.34	Person fit residual mean (SD)	-0.15 (0.91)^b^
Somatic, general	0.04	0.77	5.22	0.74	% of the 1st principal component of person residuals	28,02%^c^
Anxiety, psychic	0.20	0.89	6.44	0.6	% t tests (95% CI)	6,2 (0,04–0,1)^d^
Guilt	1.42	-0.14	8.75	0.36	**Total Item χ2**	**56.133**^**e**^
Retardation	1.47	1.66	4.02	0.85	**χ2 p value**	**0.196**^**f**^

^a^ Model fit when the value is between -2,5 and 2,5.

^b^ Perfect model fit when mean = 0 and standard deviation (SD) = 1.

^c^ Considered unidimensional when <40%.

^d^ Considered unidimensional when either less than 5% of t tests are significant, or if the lower bound of the binomial confidence interval overlaps the 5%.

^e^ Best model fit when it is a low number.

^f^ Model fit when >0.01, demonstrating the fit to the unidimensional Rasch model.

### The MDD Symptom Hierarchy

**Fig 1:**
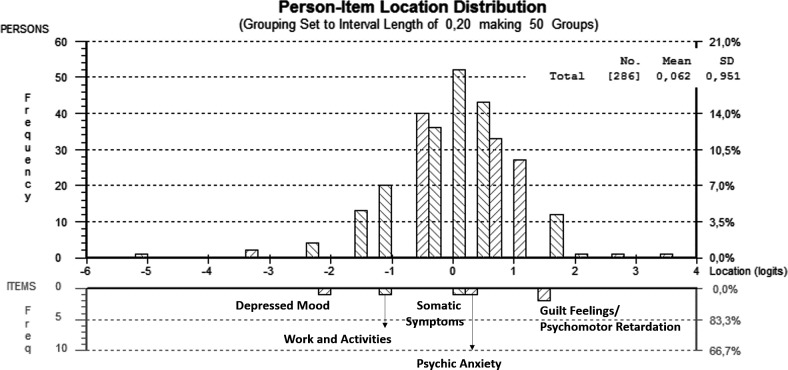
Major Depressive Disorder Signs and Symptoms Hierarchy. The person-Item map shows the severity scores for both the persons (top chart) and the items (bottom chart). The location (logits) is the measurement of the severity score (i.e., the higher is the logit, the more severe are the persons and the items). When any sign/symptom has the same Rash score of any person, it means that this person has a 50% probability to present that sign or symptom. So, any person that has a lower score of any item is less probable to present that sign or symptom; in contrast, any person that has a higher score of any item is more probable to present that sign or symptom.

The MDD symptom hierarchy is represented in Fig 1. It shows the person-item map with the location of the Rasch scores (“severity” score) of persons (top chart) and each item of HAM-D_6_ (bottom chart). The order of the severity of the melancholic features was as follows: (1) depressed mood; (2) work and interests; (3) somatic symptoms; (4) psychic Anxiety; (5) feelings of guilt; and (6) psychomotor retardation. Although the “anxiety” item was more severe than the “somatic symptoms” item, it did not show the capacity to differentiate persons in the person-item map. The same happened to the item “psychomotor retardation” and the item “guilt feelings”.

## Discussion

The present study has three main findings. The first one is that the 6 melancholic features of HAM-D_6_ are well distributed across the continuum of severity of MDD in hospitalized patients. This means that they are able to differentiate both the less and the most severe patients in this setting. The items *psychomotor retardation* and *guilt feelings* are in the top level of severity, followed by *psychic anxiety*, *somatic symptoms* and *work and activities* in the middle, and *depressed mood* at the bottom level. The second one is that, HDRS-17 does not fit to the unidimensional Rasch model and may not represent a continuum of symptoms that get worse whereas the syndrome becomes more severe, as also previously showed by Bech [12]. Finally, the third major finding is replication of Bech’s original study [31], showing that a group of 6 symptoms extracted from the HDRS-17 (HAM-D_6_) represents an unidimensional construct that clinically represents *melancholic depression in hospitalized patients*.

In the present study, we could determine through Rasch analysis the hierarchy of the six melancholic features of HAM-D_6_ in relation to MDD. Considering MDD syndrome as a continuum, *depressed mood* was the item that could better discriminate the bottom level of the MDD. On the other hand, psychomotor retardation followed by guilt feelings are the best items to discriminate the upper level of the MDD. Psychomotor disturbance is a consistent feature in differentiating melancholic and non-melancholic depression [18,48,49]. Since melancholic patients tend to be more severe [50], it is expected that they present more psychomotor changes. Our data, however, do not invalidate the theory that melancholic patients are a different group of patients [17], with differences only in a quantitative way. The item *psychomotor retardation* showed very good psychometric properties, because clinicians could precisely identify every category of the item (none of them needed to be collapsed) and could well differentiate less severe patients from the more severe ones. On the opposite pole, *depressed mood* is the central symptom of non-melancholic depression [51] and also an essential symptom in DSM system required to fulfill criteria for MDD [9].

We call attention that the item “depressed mood” is at the bottom of severity, suggesting that other symptoms continue to get worst, despite the level of the mood. This could have happened because depressed mood may work as a filter symptom, since it is a DSM-V required symptom to fulfill the diagnosis of MDD [9]. Nevertheless, we could show quantitatively that other symptoms than the mood itself must be used as clinical markers of MDD severity in a hospital unit.

The second more severe symptom item–guilt feelings–is a controversial item in the HDRS-17 (and, consequently, in HAM-D_6_), because it may measure not only guilt feelings but also psychosis [26]. A patient who scores 3 or 4 in this item is supposed to have delusions or hallucinations and that could be the explanation for how the item becomes a clinical marker of MDD severity. Nevertheless, it is interesting that, if psychosis is responsible for the severity of the item, it was less severe than psychomotor retardation, although psychotic depression is traditionally the more severe MDD subtype [5]. The finding of feelings of guilt as a severe symptom supports recent data that relates rumination with long-term prediction of MDD relapse [52].

Unidimensionality is a major requisite for a scale. According to Vellis (1991) "a unidimensional scale or a single dimension of a multidimensional scale should consist of a set of items that correlate well with each other" (p. 25) [53]. The major consequence of the unidimensionality is to justify adding the item scores together to generate a single-scale score. Although HDRS-17 is by far the most used depression scale in the literature, and its global score is the main outcome of antidepressant trials, our data points out that HAM-D_6_ but not HDRS-17 fulfill all the criteria for the unidimensional Rasch model. Although the robust t test approach to unidimensionality proposed by Smith [54] was fulfilled for both scales, only HAM-D_6_ fit to the unidimensional Rasch model. This demonstrates that only HAM-D_6_ has the property of an additive structure that enables the correct interpretation of the symptom hierarchy.

When comparing the internal consistency of both HDRS-17 and its subscale (HAM-D_6_), HDRS-17 had a slightly higher internal consistency than HAM-D_6_, but this does not mean that HAM-D_6_ is a less valid measure, since those results are highly dependent of the number of items.

The main limitation of the present study is that it is based on a sample of inpatients that do not represent the whole universe of depressive patients, and consequently the external validity of the study could be questioned. However we must remember that HDRS-17 was originally built to measure depression in inpatients diagnosed with depression [55]. Furthermore, we note that our data is very similar to that obtained by Bech [12,56] in developed countries, showing replicability, although there were some methodological differences. Furthermore, this study has the advantage of having a larger sample that found the same results even in a very different setting, suggesting that the signs and symptoms severity of MDD remain invariant in different populations. The ordering of melancholic features, correlating them with the severity of MDD, is clinically meaningful for many reasons. First, we must consider that in many contexts psychiatric diagnostic is based on a checklist symptom profile, where every symptom is given the same weight to diagnosis (except mood and anhedonia, which are considered necessary symptoms). Understanding the symptoms as a clinical marker of severity may suggest that some of them (e.g., guilt feelings and psychomotor retardation) should have a higher weight on the diagnosis of MDD, in spite of other symptoms that may be not part of the same underlying unidimensional construct [57]. Second, this could be the start point to understand how the severity of melancholia can be related to different brain areas, since different MDD symptoms does not seem to affect only one region of the brain [58]. For example, preliminary studies showed that a decreased function in the dorsolateral prefrontal cortex is associated with psychomotor retardation and apathy, while a increased function in ventromedial prefrontal cortex is more associated with increased punishment sensitivity and rumination [59]. To establish the ordering (i.e. hierarchy) of these phenomena can be helpful in understanding melancholia physiopathology.

Our data reaffirm the need for a deeper research of the essential components and of the severity indicators of the depressive disorders. We believe that this is an necessary step to the development of new classificatory systems, such as the Research Domain Criteria (RDoC)[60,61] and the 11^th^ International Classification of Diseases (WHO). In this perspective, better description of more homogeneous depressive disorders can help both the research of biological substrates and the particular understanding of the course of them.

In conclusion, we think that the understanding of MDD as a continuum of symptoms with different underlying mechanisms would better improve the understanding of the disorder and may improve perspective of treatment for patients.

## Supporting Information

S1 Data data. (XLSX)
